# rnaSeqMap: a Bioconductor package for RNA sequencing data exploration

**DOI:** 10.1186/1471-2105-12-200

**Published:** 2011-05-25

**Authors:** Anna Leśniewska, Michał J Okoniewski

**Affiliations:** 1 Poznan University of Technology, Institute of Computer Science, ul.Piotrowo 2, 60-965 Poznan, Poland; 2 Functional Genomics Center UNI ETH Zurich, Winterthurerstrasse 190, CH-8057, Switzerland

## Abstract

**Background:**

The throughput of commercially available sequencers has recently significantly increased. It has reached the point where measuring the RNA expression by the depth of coverage has become feasible even for largest genomes. The development of software tools is constantly following the progress of biological hardware. In particular, as RNA sequencing software can be regarded genome browsers, exon junction tools and statistical tools operating on counts of reads in predefined regions. The library rnaSeqMap, freely available via Bioconductor, is an RNA sequencing software which is independent of any biological hardware platform. It is based upon standard Bioconductor infrastructure for sequencing data and includes several novel features focused on deeper understanding of coverage expression profiles and discovery of novel transcription regions.

**Results:**

rnaSeqMap is a toolbox for analyses that may be performed with the use of gene annotations or alternatively, in an unsupervised mode, on any genomic region to find novel or non-standard transcripts. The data back-end may be a MySQL database or a set of files in standard BAM format. The processing in R can be run on a machine without any particular hardware requirements, and scales linearly with the number of genomic loci and number of samples analyzed. The main features of rnaSeqMap include coverage operations, discovering irreducible regions of high expression, significance search and splicing analyses with nucleotide granularity.

**Conclusions:**

This software may be used for a range of applications related to RNA sequencing by building customized analysis pipelines. The applicability and precision is expected to increase in parallel with the progress of the genome coverage in sequencers.

## Background

Massive parallel sequencing of short oligo reads has already found multiple applications in molecular biology. One of the promising novel ones is RNA sequencing, used to determine abundance of transcripts in the sample [1] - which is a more general description of gene expression profiling. The throughput of commercially available sequencers has reached the level where the depth of coverage is sufficient to measure the differences in RNA expression for the larger genomes. For example - in a typical run of ABI SOLID v4, there are 800 million reads (50 bp each). Assuming that half of these may be mapped to the known human genes, it gives 20 Gbp of coverage, which allows for more than 10 times coverage of all the Ensembl human genes. In practice, the distribution of reads coverage over the genes is very skewed.

A recent study [2] also shows good correlation of transcription measurements between RNA sequencing and microarrays even in the cases with limited number of replicate samples.

As has already happened with other technologies in molecular biology, the software development is trying to catch up with the improvements in the hardware [3]. A number of recent significant developments in the area of read mapping software [4] allow the building up of tools for both managing short reads data and for secondary analysis adapted to particular biological applications. In the first group there are ShortReads package [5], Genominator package or several commercial tools.

In the case of RNA sequencing the current approaches in secondary analysis tools are focused on three categories: genome browsers for displaying the reads over the genome [6,7], statistical tools to find significantly expressed genes and tools for predicting the transcript structure with coverage and exon junctions, such as the Tophat-Cufflinks pipeline [8,9], Scripture [10] or MapSplice [11].

The group of statistical software packages introduces the use of negative binomial distribution [12,13] of counts within genes to find the significant ones. This has solid statistical foundations and usually relies on the databases of annotations to determine the loci where the reads are counted. However, the microarrays have already demonstrated that aggregating gene expression values on the gene level or averaging of the expression of gene fragments, is often very useful but may lead to spurious results in case of non-typical transcripts [14,15].

Many of the tools may be used in a parallel computing environment, which enables publicly available cloud computing (e.g. by EBI services [16] or with Myrna [17]). However, the assumption of the rnaSeqMap library is the minimization of the computing resources needed and platform independence for the secondary analysis. Although the pipelines created with rnaSeqMap may be parallelized to multiple cores with standard R or MySQL mechanisms, they are supposed to run efficiently on a single, standalone machine. Using the pre-defined annotation of genes, transcripts and exonic regions is not taking full advantage of the predictive qualities of RNA sequencing data. The annotations can be assumed to be the real expression area boundaries, whereas the expression does not often follow the patterns frozen in the annotation databases [18].

The new Bioconductor library, rnaSeqMap, tries to overcome these limitations. This is achieved by describing the expressed regions not only by counts, but also by determining the boundaries with nucleotide precision. It may enable the exploration of RNA sequencing data using pre-defined annotations, but also complementarily in a purely exploratory way - by adjusting the findings to the expressed areas. rnaSeqMap can not only merely use the annotations, but may also confirm them, modify them or create novel ones. Managing such a massive amount of RNA sequencing data is another difficult issue. The operational memory is too small to keep the whole datasets, so it is necessary to use special mechanisms to select fragments of the data and annotations from the storage and process them. rnaSeqMap solves this issue by keeping the sequencing reads and the annotations in the same relational database.

The assumption of rnaSeqMap is to use the existing tools in the areas where they evolved into useful solutions. Its main goal is to serve as a complementary "middleware" to create analytical and discovery pipelines. Thus, it relies on pre-processed mapping of the reads to a reference genome and well known database of annotations in the back-end and on existing software for finding significantly expressed genes in the front-end. rnaSeqMap was designed to be independent of any sequencing platform, mapping software and statistical add-ons. By running it with real experimental data it was shown to be efficient at both tasks. In particular, the tests and data presented in the paper come from sequencing with an ABI SOLiD machine and mapping the colorspace reads using Bioscope.

## Implementation

### Design paradigms

rnaSeqMap has been designed according to a set of principles that turned out to be useful and efficient in exonmap - the Affymetrix exon array analysis software in Bioconcuctor and its continuation, xmapcore [15,19,20]. Those paradigms are:

• working with the genome coordinates of expression areas

• using database back-end for annotations and mapped reads

• top-down analysis - starting from a global search and getting into interesting details

• visualization of the genome by fragments

• alternative splicing analysis

• searching for the expression in potentially non-coding or non-annotated areas

rnaSeqMap uses reads mapped to the genome, characterized by a set of genome coordinates: chromosome, start, end and strand of the mapping. The option of using data from unstranded protocols is available. In this paper all the results are from a protocol with strand information. Using such minimal information about reads gives the opportunity to freely tune parameters such as mismatches or multiple targeting in the mapper software. Scripts that may be found in the installation version of rnaSeqMap are examples of how the SAM files may be trimmed to this basic set of attributes.

### Back-end modes of feeding the R objects with data

rnaSeqMap supports three modes of getting the read data: directly from BAM files (binary representation of sequencer reads), from a table in the MySQL database (where the data may be processed together with annotations) and from tables in text files.

Reading and processing of BAM files uses current Bioconductor infrastructure for processing sequencing reads: RSamtools, IRanges and GenomicRanges libraries. In case of the database back-end it is expected to follow the xmapcore database format, extended by tables for sequencing reads, junction reads and samples, with appropriate stored procedures included in the SQL scripts attached to the rnaSeqMap package.

### Data processing

In rnaSeqMap the global part of the analysis finds genomic and intergenic regions and then processes the coverage function (number of reads mapped per nucleotide) iteratively for subsequent regions. This approach allows the analyses to use a limited amount of operational memory, with nucleotide-level granularity of the findings.

The regions found as a result of the analyses and their qualities (coverage, fold change, splicing index) may be visualized using their genome coordinates - also in the areas not annotated previously to any gene or exon.

**Figure 1:**
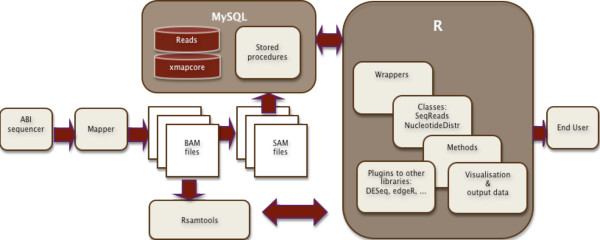
**Schema of data processing**. The flow of RNA sequencing data processing in the xmapcore database and the rnaSeqMap library.

Figure 1 presents the technical schema of the processing in the rnaSeqMap. Data generated by a sequencer are mapped to the genome annotated in the xmapcore database. The reads can be extracted from BAM files with samtools or from SAM files by an AWK script. Then the reads are imported into the MySQL *seq*_*read *table, while the *bio*_*sample *table includes the experimental setup and details of the samples. Stored procedures are used to uniformly query the extended xmapcore database and provide the data into rnaSeqMap wrappers in the R environment. The wrappers are used for building and processing objects of the classes SeqRead and NucleotideDistr. From the NulcleotideDistr object it is possible to generate distributions, summaries, visualizations or further input for higher-level statistical libraries.

### Technologies

rnaSeqMap may use MySQL database as a back-end, because Ensembl and, derived from it xmapcore, use this format. From version 5.1 onwards, MySQL engine has the partitioning mechanism which is used to partition reads in the seq_read table into sub-tables by chromosome - which speeds up searches by an order of magnitude in the case of larger genomes with multiple chromosomes.

The library has been written in R (version 2.11 or higher) and makes use, among other things, the following Bioconductor libraries: IRanges during the processing, supporting input object creation for edgeR [13] and DESeq [12]. The critical algorithms for processing nucleotide distributions were written in C for performance reasons.

### Distinctive features of rnaSeqMap

Many of the functionalities presented above are novel not only in Bioconductor. Some combinations of features are also currently unique. Such issues as the application of the same database for annotation and reads, platform and mapping independence or genome visualizations have been described above. Here we describe some other features in detail.

#### Region mining algorithm

The algorithm for finding genomic regions with the mean coverage above a defined level *μ *is an adaptation of Aumann and Lindell algorithm for mining quantitative association rules [21]. This algorithm uses two sliding windows that run across the genome, adding the coverage value of a nucleotide in every step and joining the windows under specific conditions. This results in a property of the discovered regions called irreducibility. Biological meaning and utility of the algorithm is discussed in the further sections.

#### Classes SeqReads and NucleotideDistr

To encapsulate the sequencing data in a given region, rnaSeqMap has two classes. SeqReads keeps the raw read data and may be filled in from a database or directly from a file. From an object of the class SeqReads, an object of NucleotideDistr (an S4 class) is constructed. In the data slot, it holds the distribution of the coverage function for all the nucleotides in the studied region. Then the NucleotideDistr object may be a subject to various transformations of the coverage.

#### Building blocks for analysis pipelines

The building blocks of the analytic infrastructure in rnaSeqMap may be grouped in the following categories:

• database access procedures, which are in fact wrappers for MySQL stored procedures - similarly as in xmapcore

• classes for encapsulating the sequencing reads and distributions

• functions for normalization of the coverage distributions (eg. lowess [22]), summarizing them, calculating fold change and splicing index on the nucleotide level

• Aumann-Lindell two-sliding-window algorithm implementation

• functions for finding genomic and intergenic regions in given fragments of chromosomes - to iterate the searches over them

• connectors to statistical libraries - DESeq and edgeR

• visualization - genome plots and histograms

**Figure 2:**
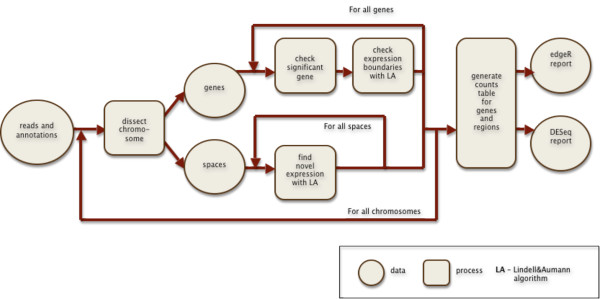
**An example of rnaSeqMap analysis pipeline**. The figure depicts data containers (circles) and processes (squares) that form the processing flow of the analysis. It iterates over chromosomes. The chromosomes are dissected into gene regions and intergenic spaces. The read counts in genes and significant novel regions of expression are then made available to the statistical analysis packages.

Those building blocks may be used to construct processing pipelines that iterate over fragments of chromosome, genes or intergenic spaces (see Figure 2).

#### Nucleotide-level splicing index

The idea of the splicing index comes from the paper [23] and in rnaSeqMap was adapted to the sequencing data defined for every nucleotide, where the coverage may differ by many orders of magnitude. The nulcleotide-level splicing index is defined as follows:

where *E*_1*n *_and *E*_2*n *_are the coverage values for a given nucleotide, while *G*_1*n *_and *G*_2*n *_are the counts of reads in the region or gene.

**Figure 3:**
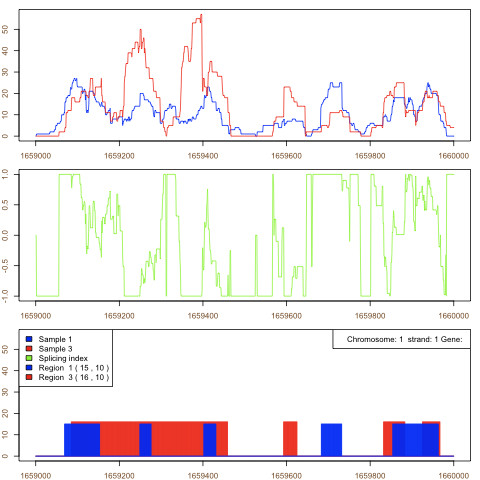
**An example of splicing index plot**. Two coverage functions on the same genome region transformed into a splicing index. The first plot presents the distribution in two samples, the second, the calculated splicing index. The third plot includes the irreducible regions of expression for both coverage functions.

Such an approach enables visualization of the splicing index on a genome plot and exploring its significant regions using the Aumann-Lindell algorithm. The example of the splicing index plot is presented on the Figure 3.

#### Discovery mode

The genome regions and their analysis may be categorized in the following way:

• gene regions, with boundaries defined according to annotations, searched for expression within the limits

• extended gene regions - to check possible expression up- or downstream from a gene and extend its boundaries

• intergenic regions - searched with the Aumann-Lindell algorithm for novel expression places

The analysis by the two latter categories may lead to discovery of novel expression regions on the genome, which may be verified with databases of ESTs and other genomic sources of evidence. This type of analysis is independent from the genomic annotation scheme.

#### Providing input data to the statistical packages

Significance analysis packages for RNA sequencing such as DESeq or edgeR require the count data in defined regions (most often in genes) as an input, then they perform analysis using binomial distributions. rnaSeqMap may generate such tables from the reads data in the database, the regions may be defined as gene boundaries or just regions that happen to be found significantly expressed in the discovery mode. In this way RNA sequencing can perform significance analysis that goes beyond microarray-style predefined regions checking.

## Results and Discussion

To evaluate the predictive performance of the software on a real sequencing dataset, we randomly selected a single strand of a human chromosome (ch 15, forward) and searched for expressed regions in 6 samples. It turned out to have 1330 genes. The number of irreducible regions of expression in genes is summarized in the Table 1. Traces of expression of coverage higher than 5 are found in ca 70% of genes, while consistent expression over 6 samples with irreducible regions can be found in ca 10% of genes.

**Table 1:** The number of genes containing irreducible regions in at least × out of 6 samples on the forward strand of human chromosome 15.

		no min support condition								support > read length					
	**X**								**X**						
***μ***		**1**	**2**	**3**	**4**	**5**	**6**	***μ***		**1**	**2**	**3**	**4**	**5**	**6**

5		915	821	750	685	619	514	5		513	349	266	220	189	134

10		776	629	537	445	363	268	10		270	190	148	126	102	81

15		527	381	289	218	162	122	15		150	103	85	75	63	55

A particular point which may be the subject of wet-lab verification is whether the irreducible regions found by the Aumann-Lindell algorithm are indeed exons. The algorithm normally needs tuning with the parameters *μ *(threshold for the coverage level) and minsup, which is here the length of a region. They have to be set according to the overall coverage in the experiment and knowledge of biological factors - such as expected exon length and the characteristics of the concentration-coverage curve. Some of this tuning may be automated by multiple runs of the algorithm across the regions with different parameters. In rnaSeqMap, the function *regionBasedCoverage*() is an example of such a procedure. It searches for irreducible regions for several values of *μ *and sets the coverage value to the maximal of them. The resulting coverage function with discrete values may be more 'human readable' and this also removes peaks of over-amplified reads and less significant local minima.

The issue of time and memory efficiency of the analyses is also important. rnaSeqMap avoids memory overloads by providing the tools to slice the genome coverage into manageable fragments, still big enough to represent even longest genes measured in many samples - and encapsulating them into objects with well-defined analytical methods. The database MySQL back-end is an engine that may be run on a single standard computer and contain a database from a complete RNA sequencing experiment, in the case of BAM files the limitation is the disc capacity.

Time of analysis is comparable to other operations in sequencing, such as preparing the libraries or mapping the reads to the reference genome. The result of a scalability tests is depicted in Additional files 1 and 2 and shows that the processing time scales linearly with the number of genomic loci and number of processed biological samples. For example, for 5 BAM files of 1.5 Gb (25 M reads) each, the processing time was less than a second per gene on a standalone machine. The proof of linear complexity of the region mining algorithm can be found in [21]

### Properties and tuning the region mining algorithm

**Figure 4:**
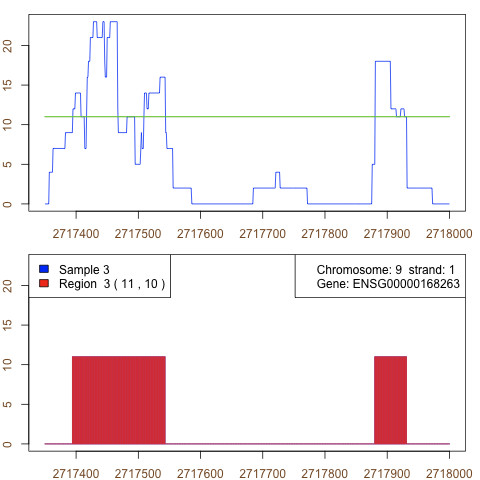
**Irreducible regions of coverage found by Aumann-Lindell algorithm**. The coverage function on the genomic region (upper plot) analysed with the Aumann-Lindell algorithm finds two irreducible regions of expression (lower plot). In the first region, the coverage happens occasionally to drop below *μ *= 11, but it keeps the property of irreducibility.

The definition of an irreducible rule in [21] follows the intuitive understanding of the expressed region in rnaSeqMap: the coverage may fall below *μ *in a fragment of an exon due to some artifacts (GC content etc), but the region itself may still have a consistent expression representation and clearly marked boundaries (see Figure 4).

According to the definition in [21], an irreducible region is one that may be split anywhere into two sub-regions with the mean value of the analyzed variable (here: coverage) remaining above the predefined *μ *level in both. In addition, it may be proven that irreducible regions always start and end with a value above *μ *[24]. Thus the Aumann-Lindell algorithm is expected to precisely find expressed regions which may be understood as exons in the biological meaning.

**Figure 5:**
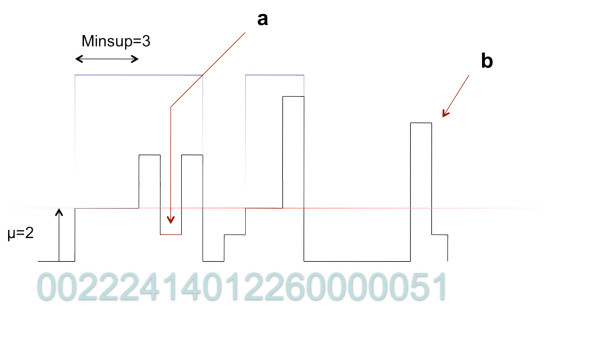
**Irreducible regions**. An example of irreducible regions found on simple numeric data. a) shows a part of the region, where the local coverage falls below *μ*, b) points out that region constraints can be used to skip the local peaks. Minsup stands here for minmal region width.

The advantages of searching for novel expression regions with Aumann-Lindell algorithms are depicted in the Figure 5 and can be summarized as follows:

• Skipping small, local drops of the coverage value, as the coverage in the region may drop locally, not violating its irreducibility

• Not overestimating the artefactual peaks, because the window algorithm does not consider them for the whole region. If the peaks are separate ones they do not fulfill the minimum support (width) condition

• Defining the boundaries of the region only for expression which is high enough, as they have to start with at least *μ *value

To tune the outcome of the algorithm several strategies may be used. Simple ones rely on a single *μ *level, that may be understood as a 'detection threshold' for the expression - often 5 is used as a threshord value for coverage. More sophisticated strategies choose *μ *as a given fraction of local minima or maxima of coverage, or apply a search with several levels *μ *of iteratively and choosing the highest as a value of coverage (*regionBasedCoverage *function).

Region mining with Aumann-Lindell rules is based upon the magnitude and irreducibility of the coverage function, while Cuffinks [9] relies mainly on exon junctions mapping. As a result the susceptibility to different types of artifacts have been observed, although the two approaches often agree in the case of well pronounced exon expression (see Additional files 3 and 4).

The lowess algorithm (locally weighted scatterplot smoothing) [22], widely used for adjusting the bias of microarrays, is used in rnaSeqMap to minimize the influence of artifacts (peaks, non-expressed small gaps) of the coverage. Applying lowess is recommended before region mining to stabilize the outcome. The effect of lowess and region mining together is shown on Additional files 5 and 6. Using lowess smoothing as a preprocessing step may possibly influence the precision of the region boundaries.

## Conclusions

Overall, the analyses performed by rnaSeqMap belong more to data mining than to statistics - as the library does searches for interesting local phenomena, without pre-assumptions, starting from a global overview and dissecting it into significant slices of expressed transcriptome. Such an approach is necessary, knowing that the existing annotations are just an approximated and constantly evolving snapshot of the real biological phenomena of transcription and alternative splicing. Although it is not a classic data mining (i.e. OLAP style), the novel features of rnaSeqMap make it different from classic genome browser and statistical tools using curated genome annotation, and is complementary to them.

The analyses performed with rnaSeq map will become gradually more precise, with the increased coverage of the RNA sequencing. This is expected, because this particular technology is currently a cutting-edge of biomolecular techniques. Thus, the applicability and utility of such an exploratory approach is expected to grow. According to [25] the RNA sequencing data are over-dispersed. There are also still a number of artifacts coming most likely from sample preparation and amplification protocols, and the closer look at the data with rnaSeqMap confirms this point of view.

## Availability and Requirements

• Project name: rnaSeqMap

• Project home page: http://www.bioconductor.org/packages/release/bioc/html/rnaSeqMap.html

• Operating systems: Windows, MacOS, Unix

• Programming language: R, C, SQL

• Other requirements: R v2.12 or higher, Bioconductor libraries v2.8 or higher, MySQL v5.1 or higher in case of using the database

• License: GPL-2

The rnaSeqMap library is available in Bioconductor from version 2.7. The MySQL database and processing may be run on any standard operating system platform. Hardware requirements do not go beyond standard desktop computers, however the amount of RAM memory limits the size of the processed objects so the size of genome fragments analyzed, and the speed of the hard drive are the main limiting factors for database operations and input BAM files.

## Authors' contributions

AL has prepared the implementation of rnaSeqMap, developed the algorithms, heuristics and the adaptation of xmapcore database, performed the quantitative experiments and helped to draft the manuscript. MO conceived the idea of the software, participated in the implementation of rnaSeqMap and drafted the manuscript. Both authors read and approved the final manuscript.

## Supplementary Material

Additional file 1 **Scalability of processing experiment**. Execution time (in seconds) of the basic operations creating SeqReads and NucleotideDistibution object, processing the coverage) for 1 to 2000 random genes and 1 to 5 BAM les, 1.5 GB (25.1 M reads) each, system time. The time is linearly scaling with the number of genes and files. Click here for file

Additional file 2 **Scalability of processing experiment**. Execution time (in seconds) of the basic operations creating SeqReads and NucleotideDistibution object, processing the coverage) for 1 to 2000 random genes and 1 to 5 BAM les, 1.5 GB (25.1 M reads) each, elapsed time. The time is linearly scaling with the number of genes and files. Click here for file

Additional file 3 **An example of Cufflinks-rnaSeq comparison**. Example of comparison of regions found by Cufflinks (Tophat mapping) and rnaSeqMap (Bioscope mapping) Click here for file

Additional file 4 **An example of Cufflinks-rnaSeq comparison**. Example of comparison of regions found by Cu inks (Tophat mapping) and rnaSeqMap (Bioscope mapping) Click here for file

Additional file 5 **An example of coverage plot smoothed by lowess**. An example of lowess use to smoothen artifacts of sequencing coverage. The original RNA-seq coverage (upper section), after lowess with f = 0.1 (middle section) and after region mining (lower section). Click here for file

Additional file 6 **An example of coverage plot smoothed by lowess**. An example of lowess use to smoothen artifacts of sequencing coverage. The original RNA-seq coverage (upper section), after lowess with f = 0.1 (middle section) and after region mining (lower section). Click here for file
