# Supplementary figures and images for: rnaSeqMap: a Bioconductor package for RNA sequencing data exploration
Source: BMC Bioinformatics. 2011 May 25;12:200. doi: 10.1186/1471-2105-12-200 (PMC3128033; doi:10.1186/1471-2105-12-200)

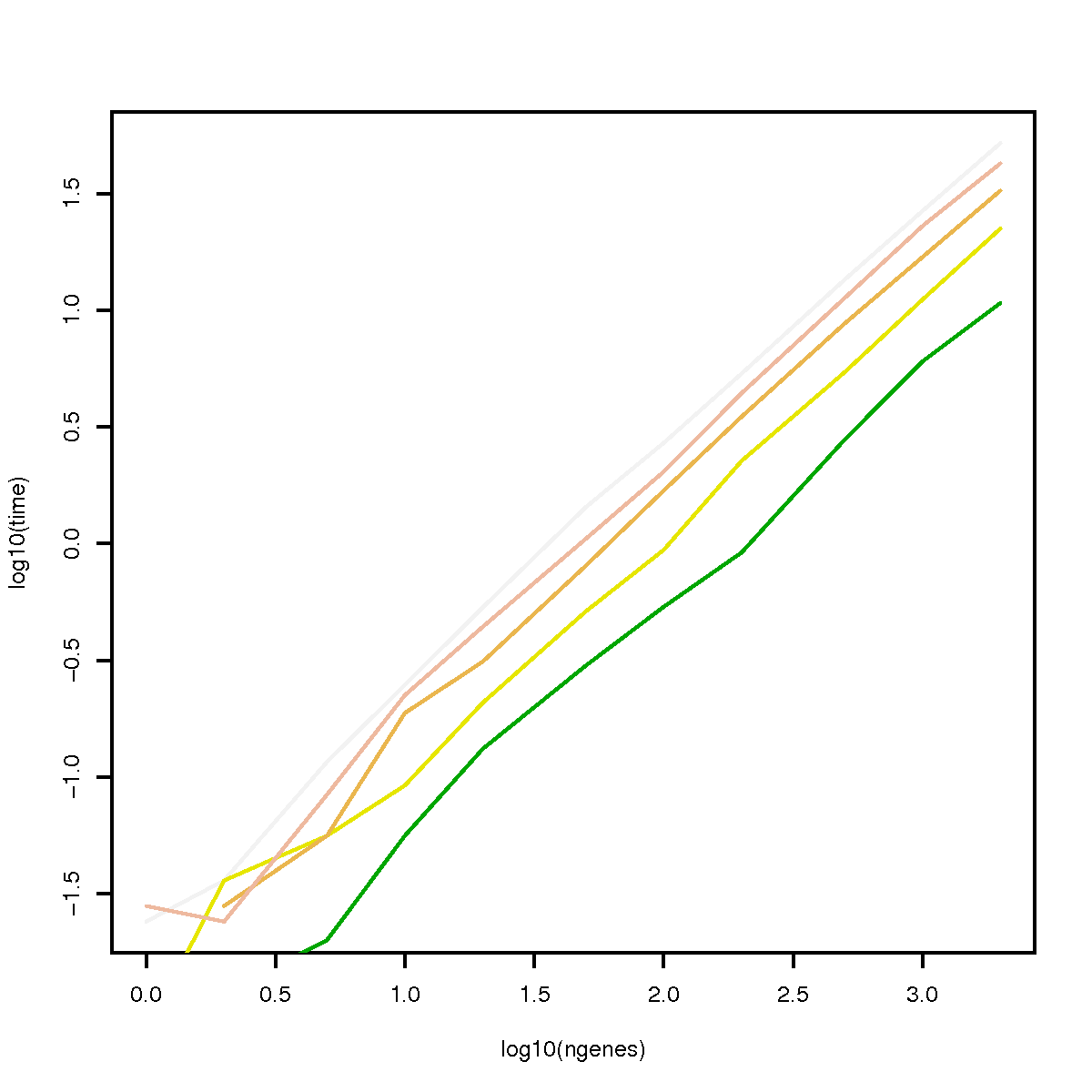

Supplement: Additional file 1 — Scalability of processing experiment. Execution time (in seconds) of the basic operations creating SeqReads and NucleotideDistibution object, processing the coverage) for 1 to 2000 random genes and 1 to 5 BAM les, 1.5 GB (25.1 M reads) each, system time. The time is linearly scaling with the number of genes and files. [file 1471-2105-12-200-S1.PNG]

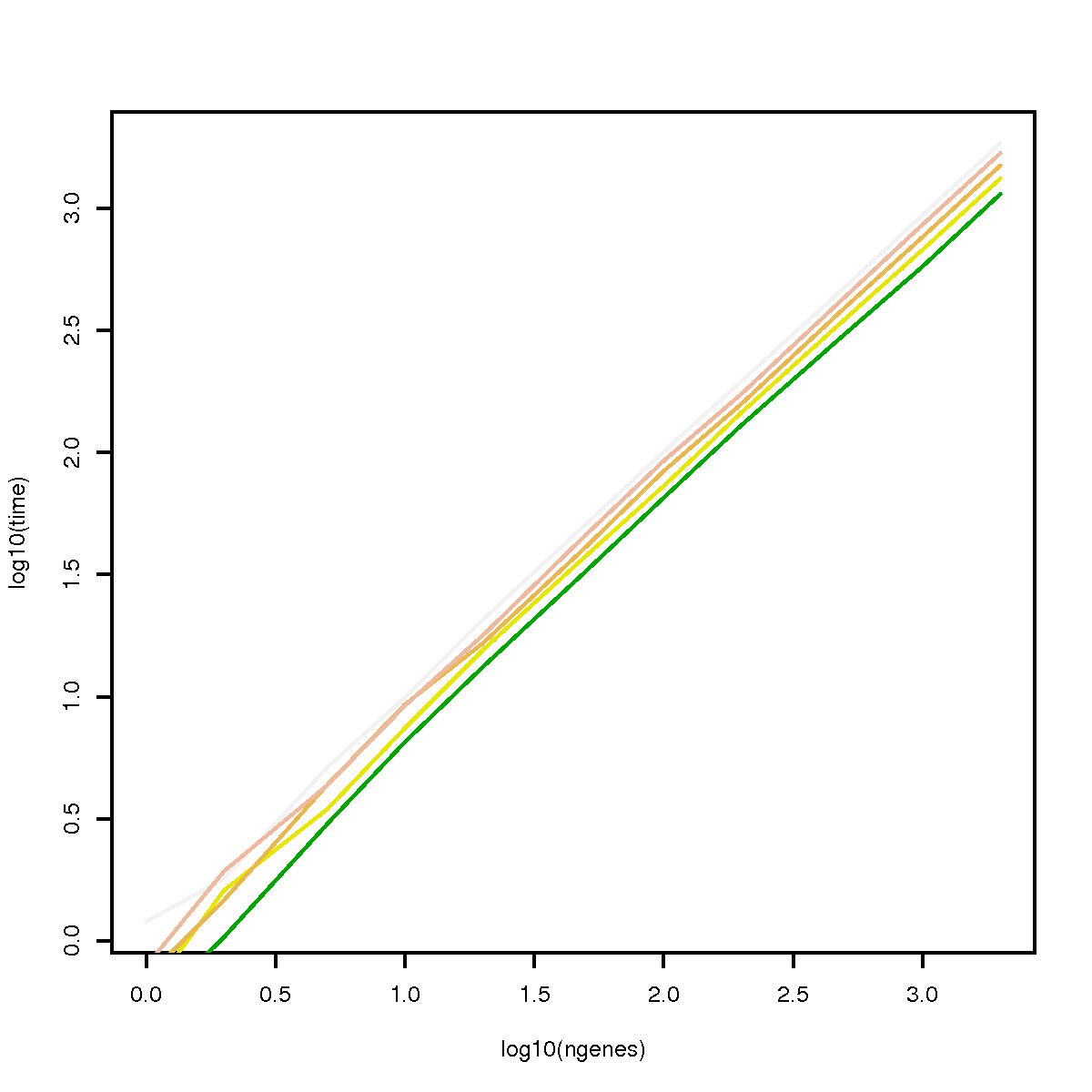

Supplement: Additional file 2 — Scalability of processing experiment. Execution time (in seconds) of the basic operations creating SeqReads and NucleotideDistibution object, processing the coverage) for 1 to 2000 random genes and 1 to 5 BAM les, 1.5 GB (25.1 M reads) each, elapsed time. The time is linearly scaling with the number of genes and files. [file 1471-2105-12-200-S2.PNG]

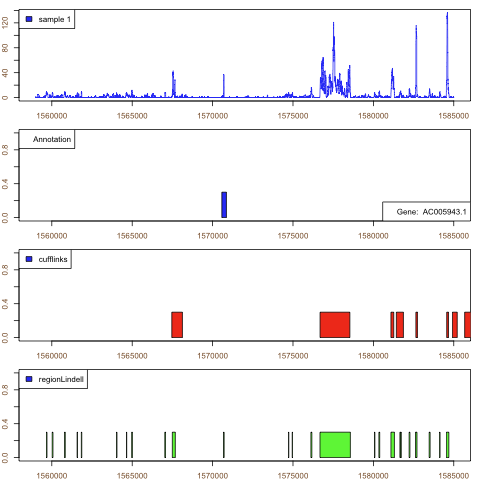

Supplement: Additional file 3 — An example of Cufflinks-rnaSeq comparison. Example of comparison of regions found by Cufflinks (Tophat mapping) and rnaSeqMap (Bioscope mapping) [file 1471-2105-12-200-S3.PNG]

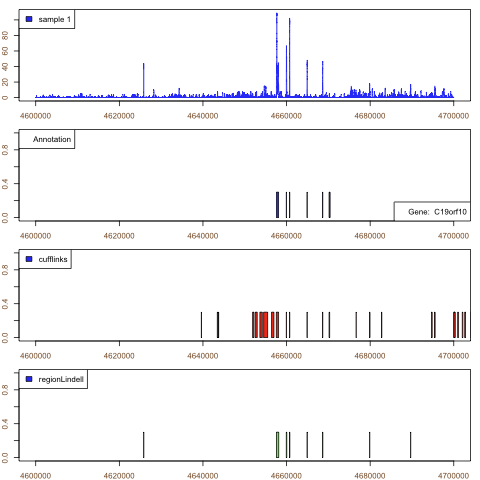

Supplement: Additional file 4 — An example of Cufflinks-rnaSeq comparison. Example of comparison of regions found by Cu inks (Tophat mapping) and rnaSeqMap (Bioscope mapping) [file 1471-2105-12-200-S4.PNG]

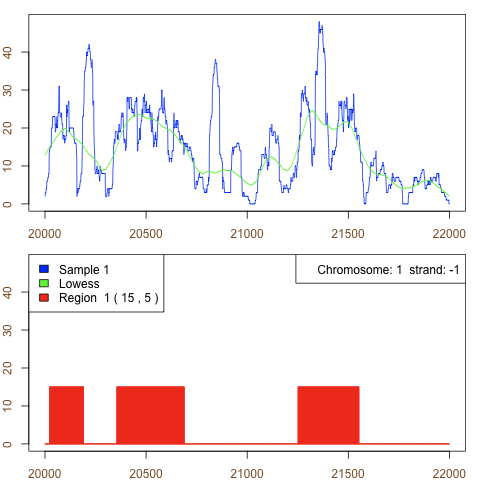

Supplement: Additional file 5 — An example of coverage plot smoothed by lowess. An example of lowess use to smoothen artifacts of sequencing coverage. The original RNA-seq coverage (upper section), after lowess with f = 0.1 (middle section) and after region mining (lower section). [file 1471-2105-12-200-S5.PNG]

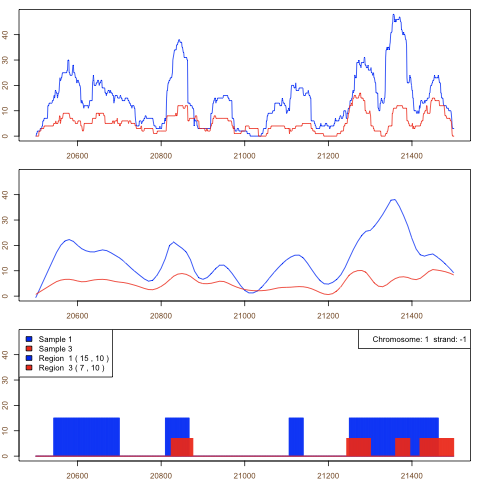

Supplement: Additional file 6 — An example of coverage plot smoothed by lowess. An example of lowess use to smoothen artifacts of sequencing coverage. The original RNA-seq coverage (upper section), after lowess with f = 0.1 (middle section) and after region mining (lower section). [file 1471-2105-12-200-S6.PNG]
